# Restaurant Menu Labeling Use Among Adults — 17 States, 2012

**Published:** 2014-07-11

**Authors:** Seung Hee Lee-Kwan, Liping Pan, Leah Maynard, Gayathri Kumar, Sohyun Park

**Affiliations:** 1 EIS officer, CDC; 2 Division of Nutrition, Physical Activity, and Obesity, National Center for Chronic Disease Prevention and Health Promotion, CDC

Many persons underestimate the calories in restaurant foods (1). Increased attention has been given to menu labeling (ML) as a way to provide consumers with point-of-purchase information that can help them reduce calorie intake and make healthier dietary choices (1–3). In 2010, a federal law was passed requiring restaurants with 20 or more establishments to display calorie information on menus and menu boards.* The regulations to implement this federal law have not been finalized, but some states and local jurisdictions have implemented their own ML policies, and many restaurants have already begun providing ML. To assess fast food and chain restaurant ML use by state and by demographic subgroup, CDC examined self-reported ML use by adults in 17 states that used the Sugar-Sweetened Beverages and Menu Labeling optional module in the 2012 Behavioral Risk Factor Surveillance System (BRFSS) survey. Based on approximately 97% of adult BRFSS respondents who noticed ML information at restaurants, the estimated overall proportion of ML users in the 17 states was 57.3% (range = 48.7% in Montana to 61.3% in New York). The prevalence of ML use was higher among women than men for all states; the patterns varied by age group and race/ethnicity across states. States and public health professionals can use these findings to track the use of ML and to develop targeted interventions to increase awareness and use of ML among nonusers.

BRFSS conducts an annual, state-based, random-digit–dialed landline and cellular telephone household survey of noninstitutionalized, civilian U.S. adults. It uses a complex multistage cluster sampling design to select a representative sample and weighting by iterative proportional fitting to adjust for nonresponse, noncoverage, and selection bias (4). A core module is administered to all BRFSS respondents and states can add topic-specific optional modules. In 2012, a ML question was offered in the Sugar-Sweetened Beverages and Menu Labeling optional module that was administered by 18 states in their combined landline and cellular survey. One state, California, was dropped from this analysis because of a high proportion of missing data for the ML question (58%). The median survey response rate for combined landline and cellular telephone respondents in the 17 states (Table 1) was 47.0% (range = 34.0%–60.4%).†

The ML question was, “The next question is about eating out at fast food and chain restaurants. When calorie information is available in the restaurant, how often does this information help you decide what to order?” Valid response options were “always,” “most of the time,” “about half the time,” “sometimes,” and “never.” The potential respondent population included 118,013 adults in 17 states. The analytic sample was limited to those who visited restaurants and noticed ML. Consequently, 10,548 respondents who said they “never noticed or never looked for calorie information” (2.2%), “usually cannot find calorie information” (0.3%), or “do not eat at fast food or chain restaurants” (6.4%) were excluded. Another 7,324 respondents (6.2%) were excluded because of missing data for the ML question. Respondents were categorized into two groups: ML users (always [11.9%], most of the time [13.7%], about half the time [8.8%], sometimes [22.8%]) and nonusers (42.7%) (Table 2). Data analyses were performed with statistical software to account for the complex sampling design. Chi-square tests were used to determine if ML use differed by age group, sex, and race/ethnicity (non-Hispanic white, non-Hispanic black, Hispanic, or non-Hispanic other races) for each state, and a p-value <0.05 was considered statistically significant. Prevalence estimates with sample sizes <50 or relative standard errors ≥30% were considered unstable and were not reported.§

In 2012, an estimated 57.3% of adults in the 17 states were ML users (Table 1). The proportion of ML users ranged from highs of 61.3% in New York and 60.2% in Hawaii to a low of 48.7% in Montana.

In the 17 states, the weighted prevalence of ML use was highest among women (66.8%) (Table 2). In each state, ML use was greater for women than men, with the highest proportion of ML female users in New York (71.0%) and Maryland (68.0%). The pattern of ML use by age group and race/ethnicity varied among the states.

## Discussion

In 2012, among adults who noticed ML information at fast food and chain restaurants, 57.3% were restaurant ML users. This is similar to the estimated 52% of BRFSS respondents in three states (Hawaii, Minnesota, and Wisconsin) who said in 2011 that they were ML users (5). In aggregate and in all states, women more often reported using ML than men. Although adults aged 35–54 years and those in non-Hispanic other racial/ethnic groups in aggregate had the highest proportion of ML users, no consistent patterns by race/ethnicity were found across states.

Among the states, some differences in ML use were noted. The prevalence of ML use in New York overall was 12.6 percentage points higher than in Montana. The reasons for differences in ML use are unclear. Factors that affect ML use, such as requirements that food service establishments display menu item calorie counts, as in New York City and several New York counties (e.g., Suffolk and Albany),¶ and promotional activities in restaurants (2) might have led to the variations across states.

What is already known on this topic?

Menu labeling (ML) can help consumers purchase items with fewer calories when eating out. An analysis of the Behavioral Risk Factor Surveillance System (BRFSS) data from Hawaii, Minnesota, and Wisconsin indicated that 52.0% of adults in the three states used ML in 2011.

What is added by this report?

In 2012, among adult BRFSS respondents in 17 states who noticed ML information at fast food or chain restaurants, 57.3% indicated that they used ML at least some of the time. Across all states, women were more likely than men to report using ML. ML use by age group and race/ethnicity varied by states.

What are the implications for public health practice?

Targeted health communication strategies might help improve awareness and use of ML and benefit adults who want to make lower calorie choices at restaurants.

Although ML use was higher among women in all of the states, ML use by age group and race/ethnicity varied across states. Previous studies reported that when calorie information is available, women were more likely to see and use this information than men (2,3,5–8). Women might perceive ML to be more useful than men (2,3). One study found women’s mean calories per purchase in restaurant chains and coffee chains decreased 18 months after implementation of ML, but men’s did not change significantly (6). The reasons for differences in ML use by age group and race/ethnicity are unknown. Further research could help identify why these disparities exist and inform targeted interventions about ML use.

The findings in this report are subject to at least four limitations. First, ML data are self-reported, and no validation studies were conducted. Second, because the BRFSS median response rate in the 17 states was 47.0% (range = 34.0%–60.4%), nonresponse bias might have affected the results. Third, because only 17 states produced usable data, the results cannot be generalized to the entire U.S. adult population. Finally, information about ML users’ food choices was not reported. Hence, data were not available to determine whether frequent or moderate ML users choose more healthful foods than nonusers.

For persons who want to reduce their caloric intake at restaurants, ML can help them select items with lower calorie content. Although research findings regarding the efficacy of ML use are inconsistent (2), some studies have found that persons who used calorie information purchased meals with about 100–140 fewer calories than those who did not see or use calorie information (6,8). Increasing appropriate use of ML might be achieved through health communication and social marketing strategies. For example, one study found that a health communication strategy that provided information on the recommended daily caloric requirement plus ML significantly reduced total calories consumed during and after the meal by 250 calories (9). Furthermore, using point-of-purchase approaches (e.g., highlighting healthful options) concurrently with ML might reinforce the selection of lower calorie, more healthful food and beverages (2). For example, ¡Por Vida!, a healthy menu initiative in San Antonio, Texas, has identified menu items that meet nutritional guidelines and lists menus and nutritional information online.** Lastly, engaging public health practitioners, restaurants, and other key stakeholders to assist in efforts to increase ML awareness and use might help patrons make more healthful food and beverage choices.

## Figures and Tables

**TABLE 1:** Fast food and chain restaurant menu labeling use among U.S. adults, by state — Behavioral Risk Factor Surveillance System, 17 states, 2012

		Menu-labeling user*	
		
State	No.†	Weighted proportion (%)	(95% CI)
Delaware	4,481	54.1	(52.1–56.1)
Georgia	5,041	56.7	(54.5–58.4)
Hawaii	6,083	60.2	(58.3–62.1)
Iowa	3,047	52.2	(49.9–54.4)
Kansas	5,265	51.3	(49.4–53.1)
Maryland	5,236	59.1	(56.7–61.6)
Minnesota	10,435	53.7	(52.4–55.0)
Mississippi	6,189	56.3	(54.4–58.1)
Montana	7,588	48.7	(47.2–50.2)
Nebraska	11,241	54.5	(53.2–55.7)
Nevada	4,086	53.9	(51.6–56.2)
New Hampshire	6,541	54.8	(53.0–56.6)
New Jersey	4,168	59.0	(56.7–61.2)
New York	4,695	61.3	(59.3–63.4)
Oklahoma	3,601	55.0	(52.8–57.2)
South Dakota	6,938	52.5	(50.6–54.4)
Tennessee	5,506	57.8	(55.9–59.6)
**Total**	**100,141**	**57.3**	**(56.6–57.9)**

**Abbreviation:** CI = confidence interval.

* Determined by responses of “always,” “most of the time,” “about half of the time,” and “sometimes” to the question, “When calorie information is available in the restaurant, how often does this information help you decide what to order?”

† Persons who reported they do not eat at fast food restaurants, could not find menu labeling, or never noticed menu labeling were excluded (8.9%).

**TABLE 2:** Proportion of fast food and chain restaurant menu-labeling users,* by state, age group, sex, and race/ethnicity — Behavioral Risk Factor Surveillance System, 17 states, 2012

	Menu-labeling user Weighted % (95% CI)†								
	
	Age group (n = 99,383)			Sex (n = 100,141)§		Race/Ethnicity (n = 96,400)			
			
State	18–34 yrs	35–54 yrs	≥55 yrs	Men	Women	White, non-Hispanic	Black, non-Hispanic	Hispanic	Other, non-Hispanic¶
Delaware	54.5 (50.1–58.8)	56.5 (53.1–59.9)	51.2 (48.7–54.1)	45.0 (41.8–48.1)	62.4 (59.9–64.9)	53.8 (51.6–56.0)	55.0 (50.6–60.5)	47.9 (37.1–58.8)	65.4 (53.7–77.2)
Georgia	52.8** (48.3–57.3)	59.8** (56.7–62.9)	56.1** (53.7–58.6)	47.9 (44.8–51.0)	64.1 (61.7–66.5)	55.7†† (53.3–58.0)	59.5†† (55.6–63.4)	48.9†† (39.8–58.0)	68.6†† (59.3–78.0)
Hawaii	64.4** (60.8–68.0)	60.0** (56.6–63.3)	57.1** (54.3–59.9)	54.1 (51.3–56.8)	66.4 (63.9–69.0)	54.9†† (51.4–58.4)	48.5†† (28.6–68.3)	64.3†† (57.4–71.2)	61.2†† (58.2–64.1)
Iowa	49.7 (44.5–55.0)	55.3 (51.6–58.9)	51.1 (48.4–53.8)	38.6 (35.3–41.9)	65.1 (62.2–68.0)	52.5 (50.2–54.8)	—§§	51.4 (37.1–65.7)	—§§
Kansas	51.6 (47.5–55.7)	53.4 (50.2–56.6)	48.9 (46.7–51.1)	40.4 (37.6–43.2)	61.9 (59.6–64.1)	51.6 (49.7–53.5)	52.7 (43.1–62.2)	48.3 (39.4–57.2)	55.5 (43.7–67.3)
Maryland	59.8** (53.8–65.8)	61.1** (57.5–64.7)	56.0** (53.1–59.0)	49.2 (45.4–53.1)	68.0 (65.1–71.0)	58.3 (55.4–61.2)	59.8 (54.9–64.7)	59.1 (46.5–71.7)	71.7 (61.9–81.5)
Minnesota	51.6 (48.8–54.5)	54.9 (52.8–57.0)	54.3 (52.5–56.1)	41.8 (39.9–43.7)	65.3 (63.6–66.9)	53.6 (52.2–54.9)	53.6 (45.7–61.5)	55.3 (47.2–63.3)	58.3 (50.9–65.6)
Mississippi	59.1 (55.0–63.1)	56.3 (53.4–59.2)	53.6 (51.5–55.7)	46.4 (43.5–49.3)	65.1 (63.0–67.3)	54.2†† (52.0–56.4)	60.4†† (57.2–63.5)	65.1†† (50.4–79.7)	50.5†† (34.9–66.2)
Montana	47.1 (43.8–50.5)	51.0 (48.4–53.6)	47.9 (45.9–49.9)	36.3 (34.2–38.4)	61.0 (59.0–63.0)	48.8 (47.2–50.3)	—§§	48.3 (36.2–60.4)	50.1 (43.8–56.5)
Nebraska	53.6 (50.9–56.3)	56.8 (54.6–59.0)	53.0 (51.3–54.8)	42.1 (40.2–44.0)	66.5 (64.9–68.1)	54.1 (52.8–55.4)	60.9 (53.7–68.1)	55.8 (49.9–61.6)	51.0 (42.0–59.9)
Nevada	54.6 (50.0–59.3)	54.5 (50.6–58.3)	52.5 (49.1–55.8)	44.2 (40.8–47.5)	63.5 (60.7–66.4)	52.2†† (49.5–54.8)	55.7†† (46.5–65.0)	53.0†† (47.8–58.3)	67.0†† (57.8–76.2)
New Hampshire	52,2 (47.4–57.0)	56.2 (53.5–59.0)	55.1 (53.0, 57.1)	43.2 (40.6–45.8)	65.8 (63.5–68.1)	54.3 (52.5–56.1)	—§§	70.2 (54.3–86.0)	56.7 (46.1–67.3)
New Jersey	57.7** (52.3–63.0)	62.5** (59.1–65.8)	55.7** (52.6, 58.8)	49.8 (46.4–53.2)	67.4 (64.7–70.2)	57.8†† (55.2–60.4)	60.8†† (54.0–67.5)	52.9†† (46.7–59.1)	74.3†† (66.6–2.1)
New York	61.3 (56.9–65.8)	63.2 (60.0–66.4)	59.2 (56.2, 62.3)	50.6 (47.5–53.7)	71.0 (68.4–73.5)	60.7 (58.4–62.9)	59.0 (52.3–65.6)	65.2 (59.9–70.6)	64.7 (55.1–74.3)
Oklahoma	58.8** (54.0–63.7)	54.8** (51.2–58.3)	51.9** (49.1, 54.6)	46.0 (42.6–49.4)	63.8 (61.1–66.5)	54.7 (52.3–57.2)	53.3 (43.8–62.7)	57.3 (48.6–65.9)	60.1 (52.1–68.2)
South Dakota	52.3 (48.8–55.7)	54.5 (51.2–57.9)	50.7 (47.7, 53.7)	39.1 (36.5–41.7)	65.3 (62.9–67.8)	52.7 (50.7–54.7)	—§§	58.0 (44.0–72.0)	52.2 (45.5–58.8)
Tennessee	62.7** (58.5–67.0)	59.7** (56.7–62.8)	51.8** (49.4, 54.2)	47.2 (44.2–50.2)	67.1 (65.0–69.2)	57.2 (55.2–59.2)	58.3 (53.3–63.4)	69.6 (52.5–86.7)	62.0 (47.0–77.0)
**Total** ¶¶	**57.1 (55.6–58.6)**	**59.4 (58.3–60.5)**	**55.1 (54.1–56.0)**	**46.9 (45.9–47.9)**	**66.8 (65.9–67.6)**	**56.2 (55.5–56.9)**	**58.9 (56.8–61.1)**	**58.2 (55.4–60.9)**	**65.0 (61.6–68.3)**

**Abbreviation:** CI = confidence interval.

* Determined by responses of “always,” “most of the time,” “about half of the time,” and “sometimes” to the question, “When calorie information is available in the restaurant, how often does this information help you decide what to order?”

† Chi-square tests were used to examine the differences in proportion of menu labeling users by age group, sex, and race/ethnicity in each state, and for the total.

§ For sex specific values, proportions significantly varied in all states; p<0.05.

¶ Non-Hispanic other race included Asian, Hawaiian or Pacific Islander, American Indian/Alaska Native, and multiracial groups.

** Within state comparison, proportions significantly varied by age group; p<0.05.

†† Within state comparison, proportions significantly varied by race/ethnicity; p<0.05.

§§ Data where the sample sizes were <50 or the prevalence relative standard errors were ≥30% were considered unstable and were not reported.

¶¶ For all tests, p<0.05.

## Footnotes

* Menu and vending machine labeling requirements available at http://www.fda.gov/food/ingredientspackaginglabeling/labelingnutrition/ucm217762.htm.

† BRFSS response rates available at http://www.cdc.gov/brfss/annual_data/2012/pdf/summarydataqualityreport2012_20130712.pdf.

§ Comparability of Data BRFSS 2012 available at http://www.cdc.gov/brfss/annual_data/2012/pdf/compare_2012.pdf.

¶ Additional information available at http://cspinet.org/new/pdf/ml_map.pdf.

** Example available at http://www.porvidasa.com.
